# Dermatologic Toxicities Associated with Tebentafusp for Uveal Melanoma: A Review of Clinical Trials and Real-World Evidence

**DOI:** 10.3390/cancers18193071

**Published:** 2026-09-22

**Authors:** Ioannis-Alexios Koumprentziotis, Aikaterini Bakella, Vasiliki Nikolaou, Dimitra Koumaki

**Affiliations:** 1 Department of Dermatology and Venereology, Medical School, National and Kapodistrian University of Athens, “A. Sygros” Hospital for Skin and Venereal Diseases, 16121 Athens, Greece; giannhskmpr@gmail.com (I.-A.K.); katebak02@gmail.com (A.B.); drviknik@yahoo.com (V.N.); 2 Department of Dermatology and Venereology, Medical School, National and Kapodistrian University of Athens, “Attikon” General University Hospital, 12462 Athens, Greece

**Keywords:** tebentafusp, uveal melanoma, dermatology, cutaneous toxicities, real-world, clinical trials

## Abstract

Tebentafusp is a first-in-class bispecific fusion protein that redirects T cells toward gp100-expressing cells and represents the first systemic treatment shown to improve overall survival in patients with HLA-A*02:01-positive metastatic uveal melanoma. As gp100 is also expressed on normal cutaneous melanocytes, skin toxicity is one of the most common adverse effects of treatment, although it remains relatively underrecognized. This narrative review summarizes the available evidence regarding tebentafusp-related cutaneous toxicity from clinical trials and emerging real-world studies. Across published cohorts, cutaneous adverse events occur in most patients receiving tebentafusp. They typically manifest within hours of the initial infusions as diffuse, frequently photodistributed erythematous eruptions accompanied by pruritus, with both frequency and severity generally decreasing with subsequent treatment. Other reported manifestations include xerosis, edema, pigmentary and hair alterations, including vitiligo-like depigmentation and poliosis, as well as less common urticarial, bullous, and photosensitive reactions. Although the development of early cutaneous toxicity has repeatedly been linked to better survival outcomes, this relationship does not appear to be independent of established prognostic variables. Accordingly, rash should currently be interpreted as a potential indicator of immune activation rather than a validated surrogate for therapeutic efficacy. Most cutaneous reactions are mild and can be effectively managed with emollients, topical corticosteroids, and antihistamines, while permanent discontinuation of therapy is uncommon. With tebentafusp becoming increasingly integrated into routine clinical practice and other gp100-targeted and bispecific T-cell–engaging therapies entering development, recognition of this characteristic dermatologic toxicity profile and early involvement of dermatology will be important for effective supportive care and continuation of treatment. Further prospective studies using standardized terminology are warranted to better define the incidence, prognostic relevance, and optimal management of these cutaneous adverse events.

## Simple Summary

Tebentafusp is a novel therapy and the first treatment shown to improve survival in patients with advanced uveal melanoma. Because it directs the immune system against a protein that is also found on normal pigment-producing cells in the skin, skin-related side effects are common, particularly rash and itching. In this review, we summarize the current evidence on these cutaneous reactions from both clinical trials and real-world clinical practice, including their frequency, presentation, management, and potential relationship with treatment outcomes. Most skin reactions are mild, occur early during treatment, and can be effectively managed without discontinuing therapy. Recognizing and appropriately treating these side effects is therefore important to help patients safely continue tebentafusp and benefit from its life-prolonging effects.

## 1. Introduction

Uveal melanoma is the most common primary intraocular malignancy in adults, yet it remains a rare tumor, with an incidence of approximately five cases per million [1,2]. It arises from melanocytes of the uveal tract, including the iris, ciliary body, and choroid, and is biologically distinct from cutaneous melanoma, characteristically harboring activating GNAQ or GNA11 mutations, BAP1 loss, and chromosome 3 monosomy, together with a low tumor mutational burden [3,4,5,6].

Although most uveal melanoma patients present with localized disease that can be controlled with surgical excision or radiotherapy, a significant number of patients eventually develop distant metastases [7,8]. Overall, metastatic uveal melanoma is associated with a relatively poor prognosis, with a median overall survival of roughly ten months according to previous reports. Unlike cutaneous melanoma, it is known that uveal melanoma does not respond equally to immune checkpoint inhibitors or BRAF/MEK targeted therapy, leading to a significant treatment gap for those affected [9,10,11].

Tebentafusp (IMCgp100; Kimmtrak) is a first-in-class immune-mobilizing monoclonal T-cell receptor against cancer (ImmTAC) [12]. It is a soluble, affinity-enhanced T-cell receptor fused to an anti-CD3 single-chain variable fragment: one arm binds a gp100-derived peptide presented by HLA-A*02:01 on melanocytes and melanoma cells, while the other engages CD3 on polyclonal T cells, redirecting them, leading to tumor lysis [12,13,14].

According to the results of the randomized phase III IMCgp100-202 clinical trial, tebentafusp became the first systemic therapy to improve overall survival in advanced uveal melanoma, leading to its approval by the US Food and Drug Administration and the European Medicines Agency in 2022 for HLA-A*02:01-positive uveal melanoma patients [15]. While offering promising outcomes, its use is associated with various toxicities, almost all explained by its mode of action through on-target T-cell activation. Such toxicities range from cytokine release syndrome to various cutaneous adverse events, most commonly appearing early during treatment and diminishing in frequency and severity with repeated dosing [16,17].

Cutaneous toxicities are among the most commonly observed organ-specific treatment-associated adverse events of anticancer therapy and, although sometimes dismissed as cosmetic, can be painful, disfiguring, and quality-of-life limiting and may lead to treatment interruption or discontinuation, affecting oncological care [18,19]. Moreover, the emergence of cutaneous toxicities has been considered to serve as a biomarker of immune activation, a paradigm first established with the use of immune checkpoint inhibitors in cutaneous melanoma [20]. In that case, the presence of vitiligo-like depigmentation and other cutaneous immune-related adverse events has been associated with improved clinical outcomes and patient survival [20,21].

Tebentafusp’s dermatologic adverse event profile reflects an on-target, off-tumor immune response against gp100-expressing melanocytes in normal skin and affects most treated patients [22]. They range from diffuse, photodistributed erythematous eruptions and generalized pruritus to hypo- or hyper-pigmented lesions and hair changes and, although usually low grade, can be severe, recurrent, and a meaningful source of symptom burden [16,22]. While our knowledge on the use of immune checkpoint inhibitors and their associated dermatologic toxicities has expanded significantly over the last years, data on cutaneous toxicities associated with tebentafusp remain scarce.

Clinical trials and real-world studies offer complementary but methodologically distinct safety data. Trials assess toxicity in selected populations under standardized protocols, with adverse events graded uniformly over fixed, often short, follow-up periods. Real-world cohorts, reflecting more heterogeneous patients and less structured, longer follow-up, can capture later-emerging events, though with greater variability in terminology and ascertainment.

As tebentafusp has recently moved from the clinical trial setting into routine oncology practice, synthesizing evidence from both clinical trials and emerging real-world cohorts is essential to define the full spectrum, time course, and optimal management of these dermatologic toxicities.

Thus, we aimed to summarize the current knowledge, arising from clinical trials and real-world practice, on their incidence, characteristic morphology, possible prognostic significance, reported treatment options and associated outcomes.

## 2. Materials and Methods

This narrative review was undertaken to summarize, evaluate, and synthesize the available current evidence on tebentafusp-associated dermatologic toxicities. A literature search was conducted using the PubMed/MEDLINE database to identify clinical trials, real-world prospective or retrospective studies, or case series/reports published until the 10 August 2026 (search date). Only publications in the English language were assessed for eligibility and relevance. The search strategy included combinations of the following keywords: tebentafusp, cutaneous toxicities, dermatologic toxicities, skin toxicities, adverse events, rash, real-world, and clinical trial. Two authors independently screened potentially eligible studies for inclusion and extracted prespecified data, including patient demographics, treatment regimen, dermatologic adverse event type, incidence, severity grade, timing of onset, management, and outcomes, using a standardized extraction template. Discrepancies in study selection or data extraction were resolved by discussion between the two authors, with adjudication by a third author when consensus could not be reached. References and citations of eligible studies were reviewed, aiming to identify additional relevant literature.

Given the known differences across the included studies in adverse-event definitions, toxicity grading criteria, dosing schedules, and follow-up duration, reported incidences were described narratively for each study rather than pooled or formally compared. This approach was chosen to characterize the pattern and range of dermatologic toxicity across clinical trials and real-world settings without implying direct statistical comparability between studies.

## 3. Tebentafusp-Associated Dermatologic Toxicities in Clinical Trials

The earliest and most granular characterization of tebentafusp-associated cutaneous toxicity derives from the trials that established the drug, in which skin-related events consistently ranked among the most frequent treatment-related adverse events and, as expected from the mechanism, affected most treated patients. Dermatologic toxicities that were described in clinical phase I–III clinical trials of tebentafusp are summarized in Table 1.

In the first-in-human phase I/II study (NCT01211262) of 84 patients with advanced cutaneous and uveal melanoma, rash of any grade affected 68% and pruritus 70%, accompanied by skin exfoliation (29%), dry skin (27%), and erythema (23%) [23]. A similar cutaneous toxicity pattern was observed in the single-arm phase II study (NCT02570308) of 127 pre-treated patients with metastatic uveal melanoma, in which rash was reported in 87%, pruritus in 67%, dry skin in 41%, peripheral edema in 31%, skin exfoliation in 22%, and erythema in 17% [24]. In the pivotal randomized phase III (NCT03070392) IMCgp100-202 trial, rash (83%) and pruritus (69–70%) were again among the most common adverse events, far exceeding the corresponding rates in the investigator’s-choice control arm (rash 27%, pruritus 21–23%) and remaining consistent in both the primary analysis and the three-year follow-up results [15,25]. Interestingly, a comparable cutaneous profile emerged in a phase 1b trial (NCT02535078), in which patients received a combination of tebentafusp with durvalumab and/or tremelimumab, with a rash being present in 67–79% and pruritus in 59–65% of patients across treatment arms and with no additional evidence of increased toxicity due to the synergistic effect of tebentafusp with immunotherapy (Figure 1) [26].

In the clinical trials, the term “rash” encompasses various morphologies of cutaneous adverse events, including erythematous, maculopapular, papular, pruritic, pustular, and vesicular eruptions, potentially understating the heterogeneity of toxicities actually encountered [15,24].

Clinical trials disclose a distinctive spectrum of skin and appendage disorders, including hypo- and hyper-pigmented lesions, that possibly arise from the destruction of gp100-expressing melanocytes and mirror the vitiligo-like changes long associated with immune checkpoint blockade in cutaneous melanoma. Vitiligo was documented in 6% of previously treated patients in the phase II trial [24] but in approximately one in five patients in the phase III (18%) and combination (15–19%) cohorts [25,26]. This incidence difference may partly reflect true biological variation but is confounded by differing follow-up durations and adverse-event ascertainment across trials.

Complementary changes included skin hypopigmentation (20%) and hyperpigmentation (17%), hair color changes and poliosis (20–27%, and up to 21% in the combination arms), alopecia (5–9%), and, less commonly, palmar-plantar erythrodysesthesia syndrome (2%) [24,25,26]. The observed pigmentary and hair toxicities were in most cases low grade and notably not amplified by the addition of agents targeting PD-L1 or CTLA-4, suggesting that they may represent an intrinsic pharmacodynamic footprint of gp100-directed T-cell activity and not an effect of immunotherapy [26]. Such observations are limited by the small sample size design along with the potential heterogeneity of clinical descriptions of lesions across the examined studies.

Regarding lesion severity, cutaneous toxicities were predominantly low grade, although the composite term rash was identified as the leading high-grade skin event, reported as grade 3 in 16–26% of patients across the monotherapy trials, whereas pruritus and the remaining dermatologic events were only rarely grade ≥3 [23,24,25]. Consistent with the observed kinetics, these events were most common during the initial step-up dosing period and became less frequent with continued treatment. Approximately 65% of patients developed a rash during weeks 1–3, decreasing to 23% by week 8, and most treatment-related adverse events in the phase III trial occurred within the first four weeks [15,24], consistent with the pooled trial safety data [16].

Tebentafusp was administered using a fixed weekly step-up regimen (20 μg on day 1, 30 μg on day 8, and 68 μg from day 15 onward) [15], a strategy designed primarily to mitigate the risk of cytokine release syndrome at treatment initiation. Notably, this toxicity kinetic does not track the dose escalation itself: cutaneous events peaked before the fixed 68 μg dose was even reached and then declined despite continued dosing at this higher, unchanged level.

This pattern potentially suggests that the timing of skin toxicity is driven less by the absolute dose administered than by the novelty of antigen exposure. Progressive depletion of the cutaneous target itself may offer a more direct explanation than adaptive immune mechanisms: declining rash frequency and severity with continued dosing has been proposed to result from gp100+ melanocyte loss, which would progressively reduce the number of available T-cell targets in the skin [27], a mechanism consistent with the declining melanocyte density observed histologically over the treatment course [28]. Contributions from T-cell adaptation, exhaustion, or homeostatic downregulation of the immune response cannot be excluded, but these have not been directly demonstrated for skin-infiltrating T cells over time and remain comparatively speculative. Whether alternative step-up schedules would meaningfully alter the incidence or severity of early cutaneous toxicity remains undefined.

Importantly, cutaneous toxicity rarely led to treatment discontinuation. No patients in the phase II study discontinued treatment because of rash [24], and only 2% of patients receiving tebentafusp in the phase III trial discontinued treatment because of an adverse event [15]. In the combination study, one patient discontinued treatment because of grade 3 rash accompanied by grade 2 generalized edema; prolonged grade 3 rash was also considered a dose-limiting toxicity [26]. No treatment-related deaths were reported across the trials. When symptomatic, rash was successfully managed with antihistamines and topical corticosteroids without interrupting treatment [24], while other skin-related adverse events were managed with standard supportive care [15].

Finally, several studies have examined whether early cutaneous toxicity carries prognostic significance, with findings that require careful distinction between simple association and independent predictive value. In the first-in-human study of tebentafusp, 69 of 84 patients (82%) who developed a rash within 21 days of the first dose had significantly longer survival than those who did not (*p* = 0.003). This association remained significant after multivariable adjustment for absolute lymphocyte count and prior anti-PD-1 therapy and was accompanied by greater induction of serum CXCL10 and a reduction in circulating CXCR3+CD8+ T cells [23]. These findings prompted a prespecified analysis in the phase III trial, where the 149 tebentafusp-treated patients who developed a rash of any grade within the first week had a substantially longer median overall survival than the 126 control patients (27.4 vs. 16.0 months; *p* < 0.001) [15]. However, this finding should be interpreted cautiously. In the same trial, the week 1 rash was not an independent predictor of overall survival after adjustment for established baseline prognostic factors, and this was confirmed at the three-year follow-up [15,25].

The trial investigators therefore advised against using rash to guide treatment decisions, since most patients eventually develop one and some patients who never develop any rash still benefit from treatment. Thus, early skin inflammation may be better viewed as a marker of on-target T-cell activation rather than a validated surrogate for clinical benefit [15]. This lack of discriminative value at the individual level highlights the need for more precise, quantitative pharmacodynamic measures. In the three-year follow-up of the phase III trial, early on-treatment reductions in circulating tumor DNA were more strongly and consistently associated with overall survival than radiographic response, suggesting that molecular measures of treatment effect may ultimately offer better individual-level predictive value than a categorical clinical sign such as rash [25].

Further insight into why this early inflammatory response functions primarily as a pharmacodynamic marker rather than a surrogate of treatment benefit has emerged from a dedicated translational analysis of trial-derived samples, integrating clinical data from the phase III study with paired on-treatment skin biopsies obtained in the first-in-human trial [29]. Histopathologic examination showed that the rash represented a T-cell–mediated interface dermatitis, characterized by a prominent infiltrate of predominantly CD8+ T cells at the dermoepidermal junction in close proximity to melanocytes, while CD4+ T cells were mainly distributed around dermal blood vessels. A rapid reduction in gp100+ melanocytes was already detectable within days of the first infusion [29]. Sequential transcriptomic analysis of these biopsies further demonstrated increased expression of cytotoxic T-cell and interferon-related immune pathways during treatment, together with coordinated suppression of melanocytic and pigmentation-associated genes. These findings provide direct in vivo evidence that the cutaneous eruption reflects an on-target, off-tumor immune response directed against gp100-expressing melanocytes in the skin [29]. Importantly, the likelihood of developing a rash was associated with baseline expression of gp100 and melanin-pathway genes as well as with melanocyte density. After adjustment for established baseline prognostic factors using propensity-score weighting, patients who developed an early rash and those who did not experienced a similar overall survival benefit from tebentafusp [29]. Taken together, these data support the concept that rash is more closely related to favorable baseline characteristics and overall immune competence than to an independent treatment-response mechanism. This helps reconcile the apparent survival association observed in earlier single-arm trials with the absence of a significant prognostic effect in the randomized analysis.

## 4. Tebentafusp-Associated Dermatologic Toxicities in Real-World Studies

Beyond clinical trial settings, an increasing number of retrospective cohorts and case series have examined tebentafusp-related cutaneous toxicity in real-world practice. Overall, these studies have largely confirmed the findings from clinical trials while providing more detailed information on dermatologic features, histopathology, and management strategies (Table 2).

The reported incidence has been consistently high and, in several studies, even higher than that observed in the registration trials, ranging from 78.8% to 89.7% in the cohorts reported [27,30,31,32]. Such high incidence rates have been suggested to arise from the longitudinal nature of the follow-up in various studies, which allowed recurrent cutaneous events throughout the treatment course to be captured. As in the clinical trials, rash and pruritus were the most frequently reported manifestations, with xerosis and facial and acral edema also commonly described, while urticarial, bullous, and photosensitivity reactions occurred less frequently [32,33,34].

Regarding morphology of cutaneous toxicities, most published series described bilateral and symmetrical erythematedematous eruptions, often with a photodistributed pattern and a clear predilection for the face and trunk. These lesions typically developed within hours of the first infusion. In contrast, pigmentary and hair changes, including vitiligo-like depigmentation, leucotrichia, and poliosis, generally appeared later, most often after several weeks to months of treatment [27,28,30,33,34,35]. In the cohort reported by Staeger et al., vitiligo-like pigmentary changes were observed in 63.6% of patients, with a median onset of 192 days, and were consistently preceded by an earlier acute tebentafusp-associated cutaneous eruption [28]. Smaller case reports have further broadened the clinical spectrum. Helf and Kähler described vitiligo- and poliosis-like depigmentation involving the skin, eyebrows, and eyelashes during the first few months of treatment in three patients [36]. Lambertini et al. reported a drug-induced photosensitivity reaction as a previously unrecognized manifestation, characterized by photodistributed erythema, edema, and pigmented macules on sun-exposed areas after the fourth treatment cycle [37]. To address the heterogeneity captured under the composite term “rash,” cutaneous morphologies are organized into standardized clinical categories in Table 3, drawing on collective clinical and histopathological descriptions from both clinical trials and real-world series.

Histopathologic findings across these cohorts largely confirmed the interface dermatitis pattern described in the original clinical trials. Biopsies from erythematous lesions typically showed focal lymphocytic interface dermatitis with a perivascular inflammatory infiltrate, often including eosinophils or neutrophils. Immunohistochemical studies further demonstrated a predominance of CD8-positive intraepidermal lymphocytes, together with reduced or absent melanocytes, supporting a mechanism of cytotoxic melanocyte destruction [30,35]. Some studies, however, identified additional histologic patterns. Zhou et al. reported a CD4-predominant interface dermatitis, which was interpreted as consistent with a type IV hypersensitivity reaction [35], while Mansilla-Polo et al. described focal subcorneal pustulation, a finding not previously reported in this setting [31]. These observations suggest that the histologic spectrum of tebentafusp-associated cutaneous toxicity may be broader than initially recognized. However, such histopathological findings are not yet established variants and arise from small series with few specimens examined. Rare bullous manifestations have also been reported. Pham et al. described superficial impetigo-like and urticarial eruptions with associated subepidermal bullae, characterized by dermal edema and perivascular eosinophils but without acantholysis or dyskeratosis, arguing against a pemphigus-like process [38].

Collectively, these findings raise the question of whether melanocyte-directed cytotoxicity alone explains the full toxicity phenotype range. Single-cell and spatial analyses demonstrated that despite an increased apoptosis-related signature, apoptotic melanocytes on cleaved caspase-3 staining remain sparse, attributed to high melanocyte BCL-2 expression and suggesting that melanocytes partially resist the cytotoxic attack against them [28]. Pigmentary changes appear to arise not only from melanocyte death but also from interferon-γ-mediated downregulation of melanin-synthesis genes (DCT, MITF), while gp100 (PMEL) itself remains stably expressed [28]. Keratinocytes and macrophages contribute to CXCL10/interferon-response upregulation and to a shift toward a pro-inflammatory M1-like state [28], pointing to a feed-forward inflammatory network beyond direct CD8+ T-cell–melanocyte interaction. Some of the observed phenotypes remain difficult to reconcile with a purely cytotoxic, melanocyte-restricted model: the CD4-predominant, hypersensitivity-type histology reported by Zhou et al. [35] and the non-acantholytic bullous eruptions described by Pham et al. [38], explicitly distinguished from a pemphigus-like process, may reflect distinct mechanisms. While melanocyte targeting almost certainly underlies the predominant erythematous and pruritic phenotypes, the full toxicity spectrum likely reflects several, only partially overlapping, processes.

The potential prognostic relationship between cutaneous toxicity and survival, initially suggested in the clinical trials, was also observed in several real-world studies, although these findings remain subject to similar interpretive limitations. Tomsitz et al. reported a substantially longer median overall survival among patients who developed skin toxicity compared with those who did not (34 versus 4 months, *p* < 0.001) [30]. Similarly, Riew et al. observed a lower rate of disease-related mortality in patients with dermatologic adverse events than in those without them (25.7% versus 75%, *p* = 0.043) [27]. This association was not consistently reproduced, however. Zhou et al. found no significant difference in disease-specific mortality between patients with and without dermatologic adverse events (23.1% versus 50.0%, *p* = 0.538), which they attributed to the small sample size and limited statistical power of their study [35]. At the level of specific clinical morphologies, Helf and Kähler noted that early depigmentation was associated with durable treatment responses in all three of their patients, paralleling the well-established association between vitiligo and treatment response seen with immune checkpoint inhibitors [36]. In the cohort reported by Staeger et al., the development of acute cutaneous adverse events was likewise associated with significantly longer overall survival. However, after adjustment for lactate dehydrogenase, age, and sex in multivariate analysis, cutaneous toxicity did not remain an independent predictor of either progression-free or overall survival. This finding is consistent with the conclusions from the clinical trials, suggesting that early skin inflammation may primarily reflect immune activation rather than represent an independent surrogate marker of treatment efficacy [28].

These observations raise the question of whether vitiligo-like depigmentation should be considered biologically and prognostically distinct from early transient rash, rather than grouped under a single composite “cutaneous toxicity” outcome. Several lines of evidence support this concept. Timing and sequence differ substantially: acute eruptions occur within hours of the first infusions, whereas depigmentation has a median onset of approximately six months and consistently follows, rather than arises independently of, an earlier acute eruption [28]. The underlying tissue-level processes also appear distinct: acute eruptions show dense T-cell infiltration and bystander keratinocyte damage, whereas depigmented lesions show comparatively reduced immune cell density at biopsy, an observation the study authors attribute to sampling from the center of established lesions, proposing that T cells may have already moved toward the advancing lesion border, though this was not directly confirmed by biopsying lesion margins [28]. Finally, limited prognostic data specific to depigmentation point the same way: Helf and Kähler observed durable responses in all three of their patients with early depigmentation [36], echoing the established vitiligo-response association seen with immune checkpoint inhibitors. Together, these findings suggest depigmentation may warrant separate consideration from transient rash in future biomarker studies.

Across most cohorts, cutaneous toxicity was generally manageable and rarely led to discontinuations. Most reactions were low grade and responded adequately to supportive measures such as emollients, topical corticosteroids, and antihistamines, with early eruptions often resolving before the next infusion [28,30]. Grade 3 reactions were relatively uncommon, occurring in approximately 17–29% of affected patients across cohorts, and were generally associated with more extensive body-surface involvement. Management included systemic corticosteroids or, in the series by Rodriguez et al., high-potency corticosteroid wet wraps and aprepitant; the latter was particularly useful in patients experiencing concurrent nausea and pruritus [27,34,35]. Permanent treatment discontinuation because of cutaneous toxicity was uncommon. In the cohort of Wang et al., only one patient discontinued treatment temporarily because of tebentafusp-associated bullous pemphigoid and was subsequently able to restart therapy safely after the reaction resolved [32]. Zhou et al. represented a notable exception, with seven patients (41.2%) discontinuing tebentafusp following the development of cutaneous adverse events [35]. Even the superficial bullous reactions described by Pham et al. resolved with topical corticosteroids without requiring interruption of treatment, suggesting that mild bullous manifestations do not necessarily warrant dose modification or discontinuation [38].

## 5. Limitations and Future Directions

Despite the increasing number of reports, several aspects of tebentafusp-associated skin toxicity remain poorly characterized. Much of the available literature consists of small retrospective series, often from single centers, with differences in terminology, follow-up duration, and assessment of toxicity severity. These limitations, together with the lack of adequately powered comparative studies, make it difficult to draw firm conclusions regarding the incidence and clinical significance of individual manifestations. Moreover, as a narrative rather than systematic review, this work did not apply formal risk-of-bias or study-quality assessment tools. The study selection process, although conducted independently by two authors, remains subject to a degree of selection bias inherent to the narrative review format. This limitation should be considered when interpreting the strength of the associations discussed.

Prospective multicenter studies using standardized dermatologic terminology and toxicity grading would be valuable to better define the frequency, timing, and potential risk factors for different cutaneous reactions. Greater attention should also be given to less common but potentially troublesome manifestations, such as bullous and photosensitive reactions, as a better understanding of their clinical and histopathological features may help guide their management. The possible prognostic relevance of particular morphologies, especially vitiligo-like depigmentation, also merits further investigation given the parallels with depigmentation associated with immune checkpoint inhibitors. Future studies could additionally explore potential biological correlates, including baseline gp100 expression, melanocyte density, and changes in circulating cytokines. However, none of these measures currently has a standardized, routinely accessible clinical assay, and each would require invasive sampling, specialized laboratory methods, and cross-cohort validation before it could aid in patient care. Unlike readily available markers such as lactate dehydrogenase, these candidates mostly represent research tools, and future work should prioritize developing standardized, ideally minimally invasive methods to assess their clinical utility.

## 6. Conclusions

Tebentafusp-related dermatologic toxicities are common and generally consistent with the drug’s mechanism of action. Across both clinical trials and real-world reports, rash and pruritus are the most frequently observed manifestations, although a broader range of findings has been described, including xerosis, edema, pigmentary and hair changes, as well as less common urticarial, bullous, and photosensitive reactions. These findings are thought to result from an on-target, off-tumor immune response against gp100-expressing melanocytes in normal skin, further supported by histopathological findings of CD8+ lymphocyte infiltration at the dermoepidermal junction together with melanocyte depletion. Although several studies have reported an association between early skin toxicity and longer survival, this relationship has not been shown to be independent of established prognostic factors; rash should therefore currently be considered primarily a marker of on-target immune activation rather than a validated biomarker of treatment efficacy.

In routine practice, early recognition and appropriate dermatologic assessment can allow symptomatic treatment to be introduced promptly and may help avoid unnecessary treatment interruptions, which is particularly relevant given the survival benefit associated with tebentafusp. As tebentafusp becomes increasingly established in clinical practice, and as other gp100-directed and T-cell–engaging therapies are developed, familiarity with their characteristic cutaneous adverse-event profiles will become increasingly important, with dermatologists playing a key role in the multidisciplinary management of these patients.

## Figures and Tables

**Figure 1:**
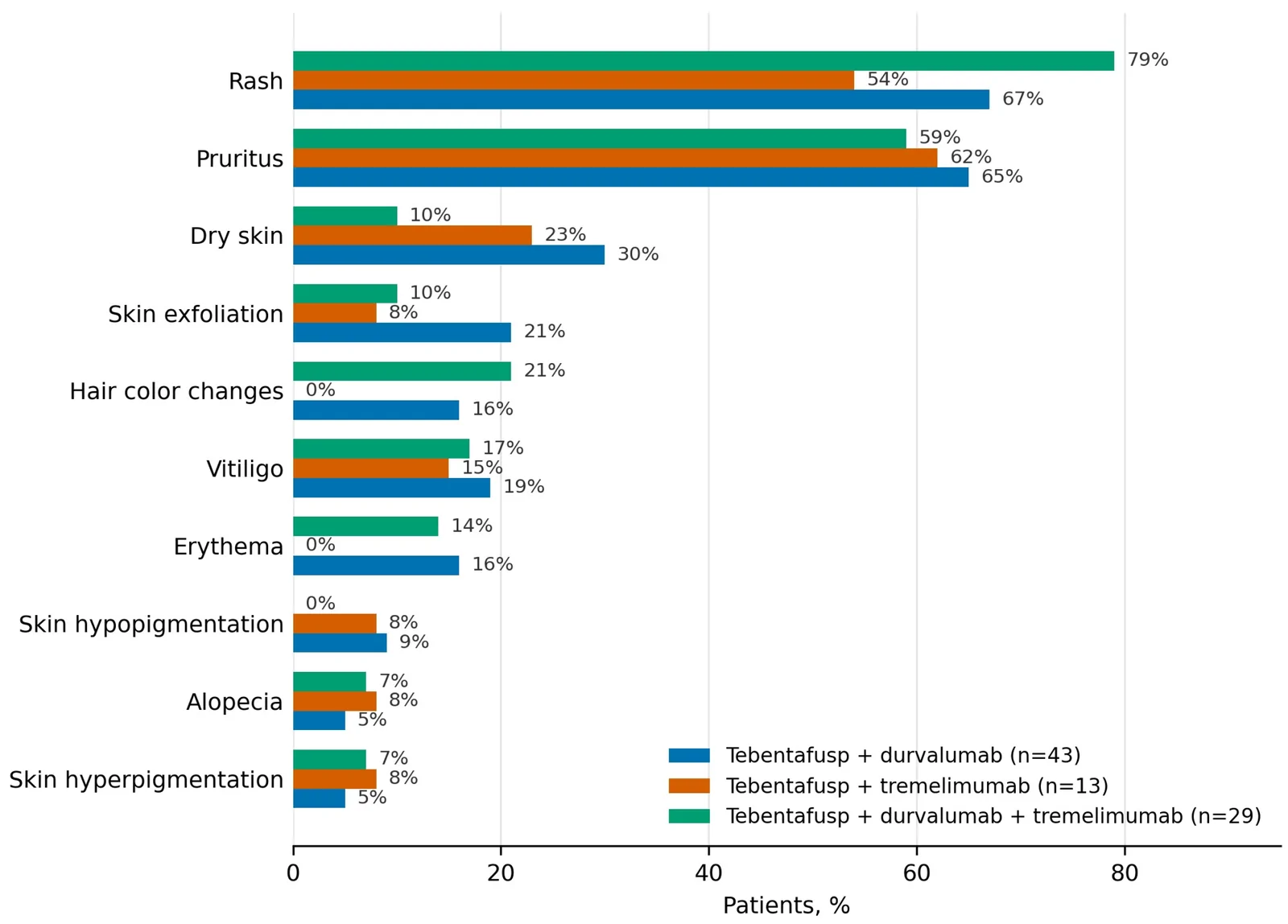
Dermatologic adverse events by treatment arm in the phase Ib combination trial (NCT02535078). Bars show the percentage of patients who experienced each cutaneous adverse event in the tebentafusp + durvalumab (*n* = 43), tebentafusp + tremelimumab (*n* = 13), and tebentafusp + durvalumab + tremelimumab (*n* = 29) arms.

**Table 1:** Tebentafusp-associated dermatologic adverse events in phase I–III clinical trials.

Author Trial Identifier	Phase	Arms	Age (Years, Median (Range))	Dermatologic Adverse Events	Total, *n* (%)	Grade ≥ 3. *n* (%)
Middleton et al. NCT01211262	I/II	Tebentafusp weekly dose escalation (Arm 1) (*n* = 66)	N/A	Rash	47 (71)	13 (20)
				Pruritus	43 (65)	0
				Skin Exfoliation	19 (29)	0
				Dry Skin	18 (27)	0
				Erythema	17 (26)	0
		Tebentafusp daily dose escalation (Arm 2) (*n* = 18)	N/A	Rash	10 (56)	7 (39)
				Pruritus	16 (89)	1 (6)
				Skin Exfoliation	5 (28)	0 (0)
				Dry Skin	5 (28)	0 (0)
				Erythema	2 (11)	0 (0)
		Tebentafusp (total) (*n* = 84)	58 yrs (25–78)	Rash	57 (68)	22 (26)
				Pruritus	59 (70)	1 (1)
				Skin Exfoliation	24 (29)	0 (0)
				Dry Skin	23 (27)	0 (0)
				Erythema	19 (23)	0 (0)
Carvajal et al. NCT02570308	II	Tebentafusp (*n* = 127)	61 yrs (25–88)	Rash	110 (87)	20 (16) N/A
				Pruritus	87 (67)	
				Erythema	22 (17)	
				Dry skin	52 (41)	
				Edema peripheral	40 (31)	
				Hair color changes	34 (27)	
				Skin exfoliation	28 (22)	
				Skin hyperpigmentation	21 (17)	
				Skin hypopigmentation	25 (20)	
				Vitiligo	7 (6)	
				Alopecia	12 (9)	
				Palmar-plantar erythrodysesthesia syndrome	2 (2)	
Hassel et al. NCT03070392	III	Tebentafusp group (*n* = 245)	64 yrs (23–92)	Rash	204 (83)	46 (19)
				Pruritus	171 (70)	11 (5)
				Dry Skin	72 (29)	0 (0)
				Erythema	59 (24)	0 (0)
				Hair color changes	50 (20)	0 (0)
				Vitiligo	44 (18)	N/A
		Control group (*n* = 111), Pembrolizumab or ipilimumab or dacarbazine	66 yrs (25–88)	Rash	30 (27)	0 (0)
				Pruritus	25 (23)	0 (0)
				Dry Skin	N/A	N/A
				Erythema	N/A	N/A
				Hair color changes	N/A	N/A
				Vitiligo	4 (4)	N/A
Hamid et al. NCT02535078	Ib	Tebentafusp + durvalumab (*n* = 43)	59 yrs (28–79)	Rash	29 (67)	N/A
				Rash maculopapular	12 (28)	
				Pruritus	28 (65)	
				Dry skin	13 (30)	
				Vitiligo	8 (19)	
				Hair color changes	7 (16)	
				Skin Exfoliation	9 (21)	
				Erythema	7 (16)	
				Alopecia	2 (5)	
				Skin hyperpigmentation	2 (5)	
				Skin hypopigmentation	4 (9)	
		Tebentafusp + tremelimumab (*n* = 13)	52 yrs (30–74)	Rash	7 (54)	N/A
				Rash maculopapular	3 (23)	
				Pruritus	8 (62)	
				Dry Skin	3 (23)	
				Vitiligo	2 (15)	
				Hair color changes	0 (0)	
				Skin exfoliation	1 (8)	
				Erythema	0 (0)	
				Alopecia	1 (8)	
				Skin hyperpigmentation	1 (8)	
				Skin hypopigmentation	1 (8)	
		Tebentafusp + durvalumab + tremelimumab (*n* = 29)	58 yrs (30–79)	Rash	23 (79)	N/A
				Rash maculopapular	3 (10)	
				Pruritus	17 (59)	
				Dry skin	3 (10)	
				Vitiligo	5 (17)	
				Hair color changes	6 (21)	
				Skin exfoliation	3 (10)	
				Erythema	4 (14)	
				Alopecia	2 (7)	
				Skin hyperpigmentation	2 (7)	
				Skin hypopigmentation	0 (0)	

**Table 2:** Tebentafusp-associated dermatologic adverse events in real-world studies.

Author	Number of Patients (Males/Females)	Age (Years, Median)	Dermatologic Adverse Events	All *n* (%)	Grade ≥ 3 *n* (%)	Additional Findings of Interest	
						Toxicity Details and Associations	Biopsy Findings
Mansilla-Polo et al.	4 (2/2)	53.5	Bilateral, symmetrical erythematedematous plaques	4 (100)	0 (0)	- Cutaneous toxicity occurred in four patients (80%), starting after the first dose (mean 17.5 h).	- Biopsy finding: Epidermis: spongiosis with focal subcorneal pustules. Dermis: interstitial infiltrates with lymphocytes, neutrophils and eosinophils.
			Pruritus	3 (75)	0 (0)		
			Palmoplantar peeling	1 (25)	0 (0)		
			Xerosis	3 (75)	0 (0)		
Riew et al.	39 (21/18)	64	Pruritus	23 (59)	N/A	- dAEs occurred beyond the fourth dose in 18 of 35 (51.4%) patients, with 4 of 31 (12.9%) continuing to experience cyclical rashes with infusions for over 250 days.	- Skin biopsies were performed in 3/35 patients, with histopathology showing interface dermatitis (2/3), spongiotic dermatitis with eosinophils (1/3), and superficial perivascular lymphocytic infiltrate (2/3).
			Rash, unspecified	11 (28)			
			Erythema	22 (56)			
			Xerosis	8 (20)			
			Facial edema	11 (28)			
			Acral Edema	3 (8)			
			Morbilliform rash	6 (15)			
			Urticaria	3 (8)			
Rodriguez et al.	6 (3/3)	51.5 years	Pruritus	6 (100)	N/A	- All patients developed pruritus, which began after their first infusion of tebentafusp. - No patient had to discontinue treatment due to cutaneous adverse events.	N/A
			Morbilliform rash	3 (50)			
			Generalized eczematous rash	1 (17)			
			Morbilliform patches	1 (17)			
Tomsitz et al. ^a^	26 (10/16) (68 lesions total)	63.5 years	**According to lesion count (*n* = 68):**		N/A	- The interval between tebentafusp dosing and first onset of skin eruption ranged from 3.5 to 7 h (median 6.5 h) in 6 patients, and in most cases (*n* = 19), skin changes were noted 20 h after the infusion. - There was no treatment discontinuation due to skin toxicities - Except for blistering changes, which took longer to heal and resulted in the formation of milia, all early skin eruptions resolved without sequelae until the next dosing 7 days later.	- 11 biopsies were taken from erythematous patches. The specimens demonstrated a mild perivascular lymphocytic infiltration with a discrete, focal interface dermatitis in all cases. In 3 of the cases, the infiltrate also contained neutrophils, and in 2 of the cases, eosinophils
			Symmetrical erythematous patches—Exanthema	57/68 (83.8)			
			Hemorrhagic macules	8/68 (11.8)			
			Urticarial lesions	5/68 (7.4)			
			Bullous lesions	1/68 (1.5)			
			Pruritus	32/68 (47.1)			
			**According to patient count (*n* = 26):**				
			Hair depigmentation	4/26 (15.3)			
			Vitiligo-like depigmented macules and patches	3/26 (11.5)			
Vitek et al.	23 (9/14)	63 yrs	Pruritus	16 (70)		- No death or treatment discontinuation was linked to adverse events.	N/A
			Rash	14 (61)			
			Palmoplantar erythrodysesthesia	4 (17)	5 (23)		
			Vitiligo-like hypo- and depigmentation of the skin	3 (13)			
			Poliosis	1 (4)			
Wang et al.	18 (11/7)	64 yrs	Rash, unspecified	13 (72)		- Of the 16 patients reporting cutaneous toxicities, 1 (6%) did not require intervention, 6 (38%) required emollient, with or without antihistamine, and 9 (56%) required TCS. - Temporary discontinuation of tebentafusp secondary to cutaneous toxicity occurred in only one patient (6%) with bullous pemphigoid. The drug was safely restarted with no further complications.	A biopsy of a pruritic bullous lesion showed subepidermal blistering with a superficial perivascular chronic inflammatory cell infiltrate and noticeable eosinophils. Direct immunofluorescence showed linear IgG and C3 deposition along the basement membrane zone, confirming bullous pemphigoid.
			Pruritus	12 (67)			
			Dry skin	8 (44)			
			Edema	5 (28)			
			Vitiligo	5 (28)			
			Leucotrichia	6 (33)	3 (17)		
			Bullous reaction	4 (22)			
			Hyperpigmentation	4 (22)			
			Skin exfoliation	4 (22)			
			Photosensitivity	3 (17)			
			Alopecia	2 (11)			
			Nail brittleness	1 (6)			
Staeger et al.	11 (8/3)	62 yrs	Diffuse erythema	9 (82)		- dAEs to tebentafusp occurred most commonly as acute skin eruption 12–48 h after the first 3 infusions. - In all cases, adAEs were transient, responsive to oral antihistamines and topical steroids, and resolved by the next infusion a week later. - Vitiligo like pigmentation disorder occurred in 7 patients, with a median onset of 192 days (range 85–275 days) following tebentafusp initiation - Development of adAEs correlated with significantly longer overall survival. In a multivariate Cox’s proportional hazards model controlling for LDH, age, and sex, adAE was not found to be an independent predictor of PFS or OS.	N/A
			Vitiligo-like pigmentation disorder	7 (64)			
			Pruritus	6 (54)			
			Facial edema	2 (18)			
			Hand edema	1 (9)			
			Macular exanthema	1 (9)			
			Maculopapular exanthema	1 (9)			
			Single fluid-filled vesicle	1 (9)			
Zhou et al.	17 (10/7)	65.7 yrs	Rash	13 (77)	5 (29)	N/A	- Two patients underwent skin biopsy: one showed CD4^+^ T-cell–mediated interface dermatitis with eosinophils and neutrophils, the other low-grade lymphocytic vasculitis with interface and eczematous change (interpreted as a type IV hypersensitivity pattern)
			Maculopapular rash	10 (59)			
			Acneiform rash	2 (12)			
			Urticarial rash	1 (6)			
			Pruritus	13 (77)			
			Edema	6 (35)			
			Xerosis	4 (24)			
			Desquamation	1 (6)			
			Hypopigmentation	1 (6)			

^a^ For Tomsitz et al. [30], dermatologic morphologies are reported as a proportion of individual lesions (*n* = 68), whereas pigmentary changes are reported as a proportion of patients (*n* = 26), reflecting the original study’s reporting. All other rows in this table report patient-level frequencies.

**Table 3:** Proposed morphological classification of tebentafusp-associated cutaneous eruptions.

Proposed Morphology	Clinical Description	Typical Onset	Representative Sources in the Literature (References)
Morbilliform/maculopapular	Diffuse erythematous macules and papules, often confluent	Early (hours to days after first infusion)	Clinical trials (rash maculopapular, 10–28%) [26]; Rodriguez et al. (morbilliform rash/patches) [34]; Staeger et al. (maculopapular exanthema) [28]; Zhou et al. (maculopapular rash, 59%) [35]
Eczematous	Erythematous, scaly plaques with spongiotic histology	Early to intermediate	Rodriguez et al. (generalized eczematous rash, 17%) [34]; spongiotic dermatitis on biopsy (Mansilla-Polo et al. [31], Zhou et al. [35])
Urticarial	Transient, pruritic wheals, often recurring with each infusion	Early, cyclical	Riew et al. [27]; Zhou et al. (urticarial rash, 6%) [35]; Tomsitz et al. (urticarial lesions, 1.5%) [30]
Pustular	Subcorneal pustules noted on histology within erythematous plaques	Early	Mansilla-Polo et al. (biopsy finding) [31]
Acneiform	Follicular papulopustular eruption	Variable	Zhou et al. (12%) [35]
Bullous	Tense or superficial blisters, including bullous pemphigoid-like presentations	Variable; can occur later in the treatment course	Wang et al. (bullous reaction, 28%, including one bullous pemphigoid case) [32]; Tomsitz et al. (bullous lesions, 1.5%) [30]; Pham et al. (case report) [38]
Photodistributed/photosensitive	Erythema and edema localized to sun-exposed areas (face, dorsal hands, V of neck)	Variable, occasionally delayed	Real-world synthesis; Lambertini et al. (case report, after 4th cycle) [37]; Wang et al. (photosensitivity, 22%) [32]
Hypopigmenting/vitiligo-like	Progressive hypopigmented macules or patches; leukotrichia, poliosis	Delayed (weeks to months); typically follows an earlier acute eruption; attributed primarily to gp100^+^ melanocyte destruction	Carvajal et al. (20%) [24]; combination trial arms (vitiligo, 15–19%) [26]; Staeger et al. (63.6%, median onset 192 days) [28]; Wang et al. (vitiligo, 28%) [32]; Vitek et al. [33]; Helf and Kähler [36]
Hyperpigmenting (post-inflammatory)	Localized or diffuse hyperpigmented macules, generally following resolution of an antecedent eruption	Typically follows resolution of an earlier inflammatory eruption; more consistent with a post-inflammatory phenomenon than direct melanocyte destruction	Carvajal et al. (17%) [24]; combination trial arms (5–8%) [26]; Wang et al. (33%) [32]
Other localized patterns	Palmoplantar erythrodysesthesia	Variable	Vitek et al. (17%) [33]

## Data Availability

No new data were created or analyzed in this study. Data sharing is not applicable.

## Abbreviations

The following abbreviations are used in this manuscript:

BAP1	BRCA1-associated protein 1
BRAF	v-Raf murine sarcoma viral oncogene homolog
CD3	Cluster of differentiation 3
CD4	Cluster of differentiation 4
CD8	Cluster of differentiation 8
CTLA-4	Cytotoxic T-lymphocyte-associated protein 4
CXCL10	C-X-C motif chemokine ligand 10
CXCR3	C-X-C motif chemokine receptor 3
GNA11	Guanine nucleotide-binding protein subunit alpha-11
GNAQ	Guanine nucleotide-binding protein G(q) subunit alpha
gp100	Glycoprotein 100
HLA-A*02:01	Human leukocyte antigen A*02:01
IMCgp100	Immune-mobilizing monoclonal T-cell receptor against cancer, gp100-directed (development code for tebentafusp)
ImmTAC	Immune-mobilizing monoclonal T-cell receptor against cancer
MEK	Mitogen-activated protein kinase kinase
PD-1	Programmed cell death protein 1
PD-L1	Programmed cell death ligand 1
